# Pattern Anlysis of Risk-Reducing Strategies in Unaffected Korean *BRCA1/2* Mutation Carriers

**DOI:** 10.3390/curroncol31110499

**Published:** 2024-11-01

**Authors:** Dabin Kim, Jai Min Ryu, Sang-Ah Han, Zisun Kim, Sung-Won Kim

**Affiliations:** 1 Department of Surgery, Soonchunhyang University Bucheon Hospital, Soonchunhyang University College of Medicine, Bucheon 14584, Republic of Korea; 129816@schmc.ac.kr; 2 Division of Breast Surgery, Department of Surgery, Samsung Medical Center, Sungkyunkwan University School of Medicine, Seoul 06351, Republic of Korea; jaimin.ryu@samsung.com; 3 Department of Surgery, Kyung Hee University Hospital at Gangdong, Seoul 05278, Republic of Korea; hansa@khu.ac.kr; 4 Department of Surgery, Daerim St. Mary’s Hospital, Seoul 07442, Republic of Korea

**Keywords:** hereditary breast and ovarian cancer syndrome, *BRCA1* gene, *BRCA2* gene, prophylactic mastectomy

## Abstract

The lifetime risk of breast and ovarian cancer increases substantially for individuals with mutations in *BRCA1/2*. The evidence indicates that *BRCA1/2* mutation carriers benefit from early cancer detection and prevention strategies. However, data on the patterns of risk-reducing interventions are lacking. This study investigated the patterns of surveillance and risk-reducing interventions among unaffected *BRCA1/2* mutation carriers. A cohort of unaffected *BRCA1/2* mutation carriers was identified from the Korean Hereditary Breast cAncer (KOHBRA) study database, and a telephone survey was conducted. The survey included questions on the incidence of new cancers, patterns of cancer (breast, ovarian, prostate, other) surveillance, chemoprevention, risk-reducing surgery, and reasons for participating in risk-reducing strategies. Between November 2016 and November 2020, 192 *BRCA1/2* mutation carriers were contacted, of which 83 responded. After excluding 37 responders who refused to participate, 46 participants (15 males, 31 females) were included in the analysis. The mean ± SD follow-up time was 103 ± 17 months (median 107, range 68~154), and the mean ± SD age was 31 ± 8 years. Ten *BRCA1/2* mutation carriers developed breast cancer, one developed ovarian cancer, and three developed other cancers. Six *BRCA1/2* mutation carriers (19.4%) underwent annual breast cancer surveillance as recommended by guidelines, while none underwent ovarian or prostate cancer surveillance. Three carriers (9.7%) used chemoprevention for breast cancer. Risk-reducing salpingo-oophorectomy was performed on only one *BRCA1/2* mutation carrier. The rates of breast/ovarian cancer surveillance, chemoprevention, and risk-reducing surgery were low among unaffected Korean *BRCA1/2* mutation carriers. Given this cohort’s relatively high risk of developing breast cancer, strategies to encourage active participation in risk reduction are needed.

## 1. Introduction

*BRCA1* and *BRCA2* are tumor suppressor genes that play a critical role in repairing DNA damage and maintaining the integrity of double-stranded DNA. Germline mutations in these genes are associated with increased risks of developing several types of cancers, including breast, ovarian, prostate, and pancreatic cancer [1,2,3].

The lifetime risks of developing breast and ovarian cancer in female *BRCA1* mutation carriers are reported to be 72% and 44%, respectively, by the age of 80. Female *BRCA2* mutation carriers’ lifetime risks are reported to be 69% for breast cancer and 17% for ovarian cancer [4]. Han et al. [5] showed that the cumulative risks of developing breast cancer by the age of 70 were 72.1% and 66.3% for female *BRCA1* and *BRCA2* mutation carriers, respectively, highlighting the significant increase in breast cancer risks for female *BRCA1/2* mutation carriers. Moreover, the cumulative risks of developing breast cancer in male *BRCA1* and *BRCA2* mutation carriers are 1% and 7–8%, respectively [6], significantly higher than the 0.1% risk observed in the average male population. Additionally, the risks of developing various cancers, such as salpinx, peritoneum, endometrium, pancreas, prostate, and colorectum, increased in *BRCA1/2* mutation carriers compared to the average population in many studies.

As a result, the NCCN (National Comprehensive Cancer Network) guidelines [7] suggest risk-reducing strategies for unaffected *BRCA1/2* mutation carriers, including cancer surveillance, chemoprevention, and risk-reducing surgery. Females with pathogenic/likely pathogenic variants of *BRCA* are recommended to undergo annual mammography and breast magnetic resonance imaging (MRI) starting at age 25 for breast cancer surveillance. For ovarian cancer risk management, risk-reducing salpingo-oophorectomy is recommended between the ages of 35 and 40 upon completion of childbearing. Male carriers are advised to undergo clinical breast exams starting at age 35 and prostate cancer screening with a digital rectal exam and prostate-specific antigen (PSA) testing starting at age 40, especially for *BRCA2* carriers. Moreover, screening for other types of cancer with increased risk, such as pancreatic cancer or melanoma, is also considered based on family history.

The mainstay of risk-reducing surgery is prophylactic bilateral mastectomy and bilateral salpingo-oophorectomy. While risk-reducing surgery can provide survival benefits, it is an invasive and irreversible method of removing an unaffected organ. In addition, the aesthetic aspects of mastectomy and iatrogenic menopause can result in a decreased quality of life. Thus, it is essential for carriers to receive sufficient genetic counseling, and healthcare providers should approach this carefully when considering risk-reducing surgery.

Chemoprevention could be considered for carriers who do not opt for risk-reduction surgery. Tamoxifen, which has been shown to reduce breast cancer incidence by 62% in unaffected *BRCA2* mutation carriers in a small study comparing its effectiveness with a placebo, is one option [8]. Additionally, oral contraceptives have been reported to reduce ovarian cancer risk by 50–55% in *BRCA1* mutation carriers and by 40% in *BRCA2* mutation carriers [9].

In spite of the benefits of the recommended risk-reducing strategies for *BRCA1/2* mutation carriers, it is currently unknown whether unaffected Korean *BRCA1/2* carriers participate in them. Therefore, this study aimed to analyze the current status of surveillance, chemoprevention, and risk-reducing surgery among unaffected Korean *BRCA1/2* carriers.

## 2. Materials and Methods

**Figure 1:**
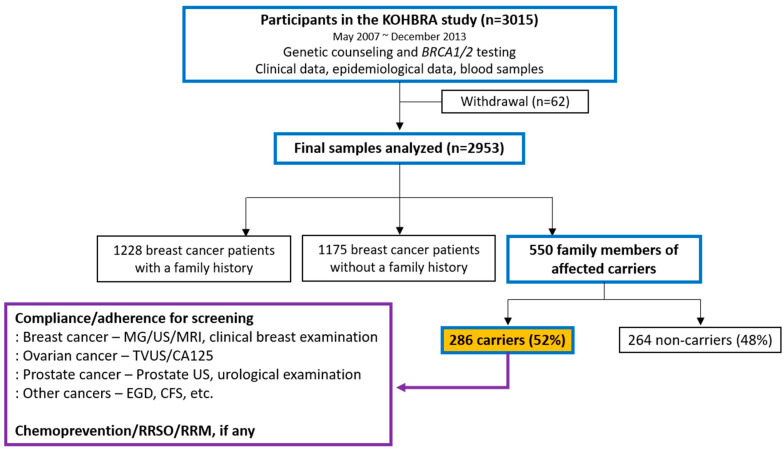
Schematic diagram of study progression.

This study was conducted on unaffected *BRCA1/2* mutation carriers with a family history of breast or ovarian cancer, identified from the Korean Hereditary Breast Cancer (KOHBRA) study database [10,11,12]. The KOHBRA study comprises 2953 *BRCA1/2* mutation carriers, and we identified family members of carriers who were affected by breast or ovarian cancer. As a result, 286 unaffected *BRCA1/2* mutation carriers were identified (Figure 1).

Due to the Personal Information Protection Act in Korea and the discontinuation of enrollment in the KOHBRA study since December 2013, obtaining follow-up data from the enrolled cohort or acquiring information from national statistics was limited initially. However, since the KOHBRA study cohort agreed that the informed consent form permitted contact for follow-up information and genetic testing at enrollment, this study was reviewed and approved by the institutional review board of Soonchunhyang University Bucheon Hospital (SCHBC 2016-08-017-001).

**Figure 2:**
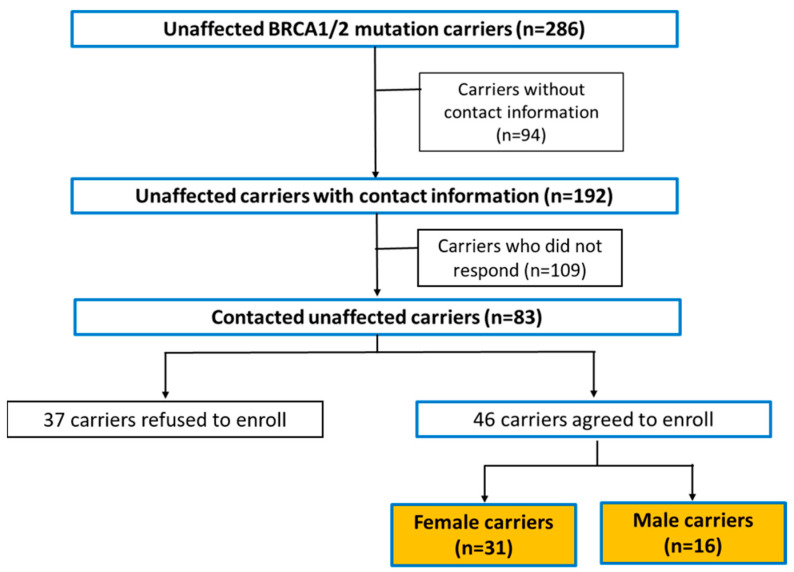
Study cohort recruitment process.

We attempted to contact 192 unaffected *BRCA1/2* mutation carriers eligible for inclusion in this study. Of these, 83 carriers successfully responded, and 37 declined to participate. Ultimately, our analysis was based on telephone survey responses from 46 carriers (31 females and 15 males) who agreed to participate in this study (Figure 2).

We conducted nine follow-up telephone surveys from August 2016 to December 2020, contacting the 46 unaffected *BRCA1/2* mutation carriers. The study period was defined as from when blood was taken for *BRCA1/2* testing to the most recent risk-reducing strategy. The telephone survey questionnaire included questions about the following:

1. Surveillance patterns for breast, ovarian, and prostate cancers;
2. Involvement in chemoprevention and the type of chemoprevention agent used;
3. Whether the carrier had undergone risk-reducing surgery and the type of surgery performed;
4. Any new onset of primary cancer;
5. The reasons for participating in risk-reducing strategies.

## 3. Results

### 3.1. Characteristics of the Participants and New Onset of Primary Cancer

The baseline participant characteristics are described in Table 1. Of the 46 unaffected *BRCA1/2* mutation carriers, the mean ± SD age was 30 ± 8 years, and the mean ± SD follow-up duration was 8.6 years (103 ± 17 months). Eleven participants had a *BRCA1* mutation (23.9%), and thirty-five had a *BRCA2* mutation (76.1%). Among the thirty-one female carriers, ten (32.3%) developed breast cancer, and one (3.2%) developed ovarian cancer. None of the male carriers developed breast or prostate cancer. The mean age of breast cancer onset among the 10 *BRCA* mutation carriers was 46 years. Detailed descriptions of the 10 *BRCA* mutation carriers are provided in Table 2.

### 3.2. Patterns of Risk-Reducing Strategies

**Figure 3:**
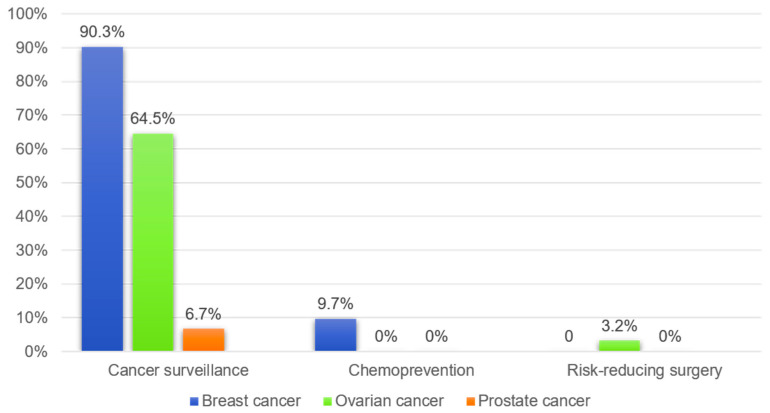
Patterns of risk-reducing strategies used by unaffected *BRCA1/2* carriers.

Of the 31 female carriers, 28 (90.3%) underwent at least one surveillance procedure for breast cancer, while 20 (64.5%) underwent surveillance for ovarian cancer. Only one (6.7%) of the fifteen male carriers underwent prostate cancer surveillance. Of the forty-six carriers, three female carriers (9.7%) were engaged in chemoprevention; all were taking tamoxifen. Additionally, one carrier (3.2%) underwent risk-reducing surgery through bilateral salpingo-oophorectomy (Figure 3).

### 3.3. Frequency and Methods of Cancer Surveillance

**Figure 4:**
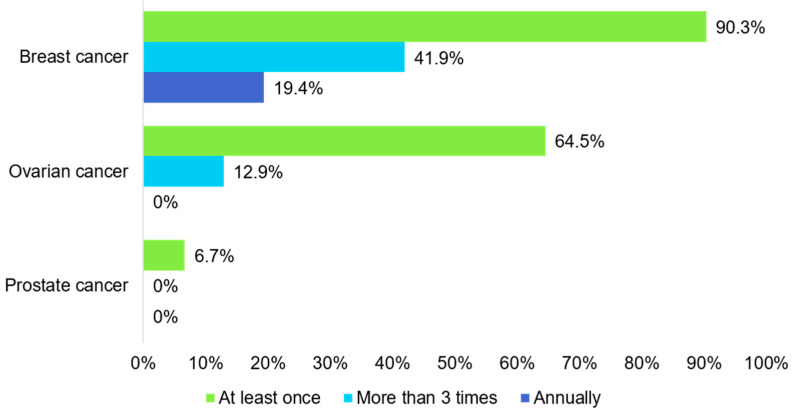
Frequency of cancer surveillance.

The frequency of cancer surveillance was assessed for carriers who reported conducting surveillance as a risk-reducing strategy (Figure 4). Of the female carriers, 28 (90%) underwent breast cancer surveillance at least once. The Korean National Cancer Screening Program provides free breast cancer screening every two years; a total of 13 (41.9%) women underwent screening more than three times during the follow-up period. However, only six (19.4%) carriers had annual breast examinations as recommended by the NCCN guidelines. Among the female carriers, twenty (64.5%) underwent ovarian cancer surveillance at least once, and four (12.9%) did so more than three times, with none undergoing annual surveillance. Only one male carrier had a prostate examination for cancer surveillance. Mammography, breast ultrasounds, and clinical breast examinations were the most common methods used, and four carriers underwent annual breast MRIs (Table 3). Additionally, all four carriers who underwent ovarian cancer surveillance more than three times had gynecological examinations and CA-125 measurements, and three of them received transvaginal ultrasonography.

### 3.4. Reasons for Participating in Risk-Reducing Strategies

**Figure 5:**
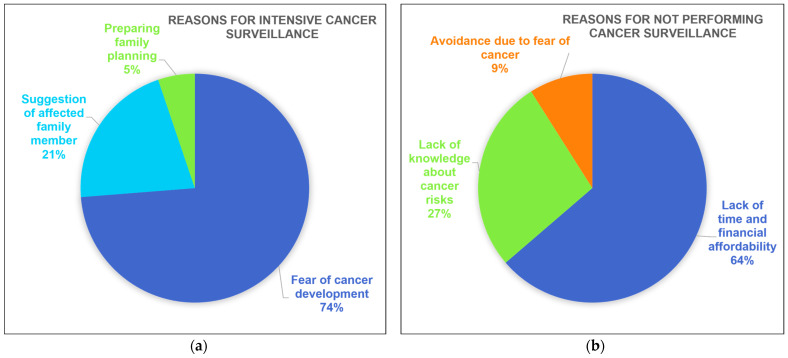
Reasons for participating in risk-reducing strategies: (**a**) reasons for participating in intensive cancer surveillance; (**b**) reasons for not participating in cancer surveillance.

Among the female carriers who carried out breast cancer surveillance at least every 1–2 years, 14 (73.6%) responded that their participation was motivated by the fear of breast or ovarian cancer, 4 (21.0%) replied that they were persuaded by family members who had already developed cancer, and 1 (5.3%) stated that it was to prepare for family planning. On the other hand, female carriers who did not undergo cancer surveillance cited reasons such as a shortage of time or financial budget (n = 7, 63.6%), insufficient knowledge about cancer risks (n = 3, 27.2%), and avoidance due to fear of cancer (n = 1, 9.0%) (Figure 5).

## 4. Discussion

This study aimed to investigate the patterns of cancer risk-reducing strategies recommended for unaffected *BRCA1/2* mutation carriers with higher risks of breast and ovarian cancer. The high incidence rate of breast cancer in female carriers (32.3%) is concerning, particularly given the young mean age of 30 years. However, less than half of the carriers (41.9%) underwent breast examinations at least once every 2–3 years, and less than 20% underwent annual examinations.

Hong et al. [13] conducted a study on breast cancer screening rates among Korean women participating in the National Cancer Screening Program. They found that the screening rate had increased, reaching 63.1% in 2018; similarly, the National Health Insurance Service reported breast cancer screening rates of 63.2% in 2017 and 64.6% in 2021 [14]. The lower breast examination rate among *BRCA1/2* mutation carriers compared to the general population is concerning, as these individuals have a significantly higher risk of developing breast cancer. This discrepancy underscores the importance of targeted recommendations and interventions specifically for *BRCA1/2* mutation carriers to ensure they understand the necessity of regular breast examinations and adhere to appropriate screening guidelines.

The NCCN guidelines [7] and the Korean Clinical Practice Guideline for breast cancer [15] recommend annual mammography and breast MRIs for female *BRCA1/2* mutation carriers. These guidelines suggest using both mammography and MRIs as screening methods for high-risk patients because breast MRIs have a higher sensitivity and lower specificity than mammography [16,17]. However, breast ultrasonography is not recommended for cancer screening, as it has not been proven to offer additional benefits in detecting breast cancer compared to mammography and MRI [18]. Despite these recommendations, our data showed that all female *BRCA1/2* carriers who underwent breast cancer surveillance used breast ultrasonography (n = 13), while only a small number (n = 4) used breast MRIs. One reason for this discrepancy could be the lack of health insurance coverage for breast MRIs in Korea for *BRCA1/2* mutation carriers, which may result in significant out-of-pocket expenses for those who choose to undergo annual surveillance. Additionally, the study participants showed a relatively young average age (mean 30 years), which could mean that annual breast MRIs were less affordable for them, leading to a lower percentage of carriers receiving the recommended screening. Furthermore, younger carriers may have fewer comorbidities compared to older individuals and may be less aware of and attentive to cancer risks.

A significant finding of this study is the discrepancy in the rates of cancer surveillance among those who underwent surveillance once, more than three times, and annually. Understanding the underlying reasons for this variation is crucial. Barriers such as financial constraints, lack of awareness, and accessibility issues need to be addressed to improve compliance with recommended surveillance strategies. Furthermore, psychological factors may play a significant role in the decision to engage in risk-reducing strategies. Among carriers who did not undergo cancer surveillance, fear of cancer was a reason for avoidance, in addition to other factors such as lack of knowledge, lack of time, and financial constraints. Considering that about 30% of the enrolled carriers developed breast cancer, these findings emphasize the need for increased awareness about breast cancer surveillance and providing more affordable screening options for *BRCA1/2* mutation carriers. Proactive measures, such as easing financial burdens through expanded health insurance coverage, enhancing educational initiatives to emphasize the importance of the early detection and prevention of cancer, raising awareness of the increased cancer risks linked to *BRCA1/2* mutations, and strengthening access to and utilization of genetic counseling services, are required to encourage continuous and comprehensive surveillance among *BRCA1/2* mutation carriers. 

Bilateral mastectomy and salpingo-oophorectomy are the centerpieces of risk-reducing surgery for unaffected carriers. Bilateral mastectomy has been reported to reduce breast cancer risk by more than 90% in high-risk populations and *BRCA1/2* mutation carriers, although the evidence regarding its survival benefits is lacking [19,20]. In contrast, bilateral salpingo-oophorectomy has been shown to reduce the risk of ovarian cancer by 80% and the risk of breast cancer by 50% in *BRCA2* mutation carriers, and it has been proven to have a survival benefit [21,22,23,24,25]. Based on this, genetic testing and bilateral salpingo-oophorectomy for the high-risk group of *BRCA1/2* mutation carriers have been included in the Korean Health Insurance Service since 2013, increasing the proportion of carriers who have undergone risk-reducing surgery from 31.6% to 46.3% [26]. However, bilateral salpingo-oophorectomy cannot be performed on women who have plans for childbearing and can lead to other morbidities such as osteoporosis or cardiac disease due to iatrogenic menopause. Therefore, careful counseling and adequate information must be provided to candidates to determine whether and when to undergo surgery.

According to previous reports, male carriers do not tend to undergo genetic testing; unaffected men underwent genetic testing for breast and ovarian cancer at one-tenth the rate of women, and patients with prostate cancer were less likely to undergo genetic testing (1%) compared to patients with breast and ovarian cancer (52.3%) [27]. However, male carriers of *BRCA1/2* mutations carry a significant burden of cancer risk, which is comparable to female carriers. Male *BRCA2* mutation carriers are recommended to undergo prostate cancer surveillance through measuring PSA, digital rectal exams, and prostate USG over 40 years of age, and this could also be considered for *BRCA1* mutation carriers. In our study, the mean age of male carriers was 28 years, and only one male carrier (6.7%) among the fifteen unaffected male *BRCA1/2* carriers was above 40, which is the age recommended for prostate cancer surveillance in the guidelines. Therefore, it is difficult to conclude whether male carriers participate in risk-reducing strategies. Considering that *BRCA1/2* mutation carriers have a significantly increased risk of prostate cancer and that prostate cancer in *BRCA1/2* mutation carriers is known to be more aggressive with a worse prognosis [28,29,30], it is crucial to emphasize the importance of prostate cancer surveillance to male carriers.

Lee et al. [26] analyzed the risk-reducing strategies used by both affected and unaffected *BRCA1/2* mutation carriers in Korea. They found that older age was a significant factor associated with risk-reducing management among affected carriers with breast cancer, and bilateral salpingo-oophorectomy was preferred over mastectomy as a risk-reducing procedure in both affected and unaffected carriers. In Lee’s study, 50% of unaffected carriers underwent cancer surveillance, and the percentages of carriers who underwent risk-reducing surgery or chemoprevention were 29.1% and 20.2%, respectively. In comparison, our data revealed that 41.9% of carriers underwent cancer surveillance examinations more than three times, which was comparable to Lee’s findings. However, only 3.2% of the carriers underwent risk-reducing surgery and 9.7% underwent chemoprevention in our study, which was significantly lower than in Lee’s data. This discrepancy might be due to the fact that the mean age of Lee’s study group was higher than ours and might have included a higher proportion of carriers who had already completed their pregnancy plans. Additionally, older individuals could be more aware of their cancer risks, leading to a higher percentage of carriers who had risk-reducing surgery or chemoprevention.

DiSilvestro et al. [31] investigated the risk-reducing strategies for ovarian cancer used by 104 female carriers of the *BRCA1/2* mutation in the northeast United States. They reported that almost all (97%) of the carriers were offered risk-reducing bilateral salpingo-oophorectomy, while only half were offered chemoprevention with oral contraceptive pills and ovarian cancer screening. However, 88% and 81% of the carriers who were offered oral contraceptive pills and ovarian cancer screening, respectively, were found to be taking the pills and undergoing screening. This demonstrates a higher proportion of participation in risk-reducing strategies than was observed in our study. These findings highlight the importance of providing education and suggesting risk-reducing strategies to *BRCA1/2* mutation carriers. 

In a study covering the risk-reducing strategies used by unaffected *BRCA1/2* mutation carriers in the Western hemisphere [32], the percentage of carriers who chose to have a risk-reducing bilateral mastectomy or bilateral salpingo-oophorectomy varied from 0 to 54% and from 13 to 78%, respectively. In contrast, our study revealed that 0% (n = 0) and 3.2% (n = 1) of Korean carriers underwent mastectomy and salpingo-oophorectomy, which is significantly lower than the Western study. Lee et al. [26] also reported that 0% and 29% of unaffected carriers underwent bilateral mastectomy or salpingo-oophorectomy. According to a domestic study, the breast and ovarian cancer risks of *BRCA1* mutation carriers up to the age of 70 were 72.1% and 24.6%, respectively, and 66.3% and 11.1%, respectively, in *BRCA2* mutation carriers [5]. Despite the slight difference in cancer risks, the immense difference in the percentage of carriers who had risk-reducing surgery could be affected by age disparities between the studies, health insurance coverage, and cultural differences regarding body image and breast or ovarian preservation.

This study aimed to analyze the patterns of cancer risk-reducing strategies used by unaffected *BRCA1/2* mutation carriers with a family history of breast or ovarian cancer who were included in the KOHBRA study. There are a few limitations to our study: a small number of carriers were included, which may lower the applicability of our study. Also, the relatively young age of the enrolled carriers may have meant that they had a lower cancer development risk and access to the healthcare system, leading to selection bias. These factors may make it challenging to generalize our findings to the larger Korean population. Additionally, there may be an inherent recall bias due to the survey-based approach. 

Nevertheless, this study is significant as it identifies the actualities of implementing risk-reducing strategies with real-world clinical data and a relatively long follow-up time (8.6 years). Another strength of this study is that it is the first in Korea to actively follow up with healthy *BRCA* carriers and verify the incidence rates of breast and ovarian cancers. This proactive approach provides valuable insights into the real-world implementation of risk-reducing strategies and their outcomes in a Korean context. By tracking the health outcomes of these carriers over time, this study highlights the practical challenges and successes of applying preventive measures in a clinical setting, thereby informing future guidelines and interventions to better support *BRCA* mutation carriers in Korea.

## 5. Conclusions

In conclusion, this study identified significant gaps in adherence to the recommended risk-reducing strategies among unaffected *BRCA1/2* mutation carriers. Despite the limitations of a small sample size and the young age of enrolled carriers, addressing these gaps through enhanced educational initiatives, financial support, and improved access to genetic counseling is crucial to better manage cancer risks in this high-risk population.

## Figures and Tables

**Table 1:** Baseline participant characteristics.

Characteristics	Value
**Number of participants**	46 (100%)
Male (n, %)	15 (32.6%)
Female (n, %)	31 (67.4%)
**Age (years) at blood test**	
Mean ± SD	30 ± 8
Median (range)	29 (20–58)
**Follow-up duration (months, mean ± SD)**	103 ± 17
Male	97 ± 16
Female	106 ± 17
***BRCA* mutation**	
*BRCA1* (n, %)	11 (23.9%)
*BRCA2* (n, %)	35 (76.1%)
**Development of breast cancer in females (n, %)**	10 (32.3%)
**Development of ovarian cancer in females (n, %)**	1 (3.2%)
Development of other cancers * (n, %)	3 (6.5%)

* The other cancers developed included two thyroid cancers and one orbital cancer.

**Table 2:** Characteristics of ten *BRCA* mutation carriers who developed breast cancer.

Age at Blood Test	Age at Breast Cancer Diagnosis	*BRCA 1/2*	Breast Cancer Surveillance			Development of Ovarian Cancer	Ovarian Cancer Surveillance		
			At Least Once	More Than 3 Times	Annual		At Least Once	More Than 3 Times	Annual
41	45	*BRCA1*			Yes	No		Yes	
58	65	*BRCA2*		Yes		No	Yes		
31	40	*BRCA2*	Yes			No	Yes		
45	55	*BRCA1*			Yes	No	Yes		
40	47	*BRCA1*		Yes		No	Yes		
34	44	*BRCA2*		Yes		No	Yes		
33	42	*BRCA2*	Yes			No	Yes		
40	48	*BRCA2*	No			No	No		
36	44	*BRCA1*			Yes	No		Yes	
27	34	*BRCA2*		Yes		No	Yes		

**Table 3:** Breast and ovarian cancer surveillance methods.

Breast Cancer Surveillance (n = 13)	
Breast self-exam	4
Clinical examination by physician	13
Mammography	13
Breast USG	13
Breast MRI	4
**Ovarian cancer surveillance (n = 4)**	
Gynecological exam	4
Transvaginal USG	3
CA 125	4

## Data Availability

The data presented in this study are available on request from the corresponding author due to the participants’ privacy.

## References

[1] Yun M.H., Hiom K. (2009). Understanding the functions of BRCA1 in the DNA-damage response. Biochem. Soc. Trans..

[2] Cipak L., Watanabe N., Bessho T. (2006). The role of BRCA2 in replication-coupled DNA interstrand cross-link repair in vitro. Nat. Struct. Mol. Biol..

[3] Friedenson B. (2005). BRCA1 and BRCA2 pathways and the risk of cancers other than breast or ovarian. MedGenMed.

[4] Kuchenbaecker K.B., Hopper J.L., Barnes D.R., Phillips K.A., Mooij T.M., Roos-Blom M.J., Jervis S., van Leeuwen F.E., Milne R.L., Andrieu N. (2017). Risks of Breast, Ovarian, and Contralateral Breast Cancer for BRCA1 and BRCA2 Mutation Carriers. JAMA.

[5] Han S.A., Park S.K., Ahn S.-H., Son B.H., Lee M.H., Choi D.H., Noh D.-Y., Han W., Lee E.S., Han S.K. (2009). The Breast and Ovarian Cancer Risks in Korea Due to Inherited Mutations in BRCA1 and BRCA2: A Preliminary Report. J. Breast Cancer.

[6] Tai Y.C., Domchek S., Parmigiani G., Chen S. (2007). Breast cancer risk among male BRCA1 and BRCA2 mutation carriers. J. Natl. Cancer Inst..

[7] National Comprehensive Cancer Network (2024). NCCN Clinical Practice Guidelines in Oncology: Genetic/Familial High-Risk Assessment: Breast, Ovarian, and Pancreatic, Version 3. https://www.nccn.org/professionals/physician_gls/pdf/genetics_bop.pdf.

[8] Eisen A., Weber B.L. (2001). Prophylactic mastectomy for women with BRCA1 and BRCA2 mutations--facts and controversy. N. Engl. J. Med..

[9] McLaughlin J.R., Risch H.A., Lubinski J., Moller P., Ghadirian P., Lynch H., Karlan B., Fishman D., Rosen B., Neuhausen S.L. (2007). Reproductive risk factors for ovarian cancer in carriers of BRCA1 or BRCA2 mutations: A case-control study. Lancet Oncol..

[10] Han S.A., Park S.K., Ahn S.H., Lee M.H., Noh D.Y., Kim L.S., Noh W.C., Jung Y., Kim K.S., Kim S.W. (2011). The Korean Hereditary Breast Cancer (KOHBRA) study: Protocols and interim report. Clin. Oncol. (R. Coll. Radiol.).

[11] Kang E., Kim S.W. (2013). The korean hereditary breast cancer study: Review and future perspectives. J. Breast Cancer.

[12] Kang E., Seong M.W., Park S.K., Lee J.W., Lee J., Kim L.S., Lee J.E., Kim S.Y., Jeong J., Han S.A. (2015). The prevalence and spectrum of BRCA1 and BRCA2 mutations in Korean population: Recent update of the Korean Hereditary Breast Cancer (KOHBRA) study. Breast Cancer Res. Treat..

[13] Hong S., Lee Y.Y., Lee J., Kim Y., Choi K.S., Jun J.K., Suh M. (2021). Trends in Cancer Screening Rates among Korean Men and Women: Results of the Korean National Cancer Screening Survey, 2004-2018. Cancer Res. Treat..

[14] National Health Insurance Service National Health Screening Statistical Yearbook 2022. https://www.nhis.or.kr/nhis/together/wbhaec07000m01.do?mode=view&articleNo=10840505&article.offset=0&articleLimit=10.

[15] Korean Breast Cancer Society (2023). The 10th Korean Clinical Practice Guideline for Breast Cancer. https://www.kbcs.or.kr/html/?pmode=recommendation.

[16] Le-Petross H.T., Whitman G.J., Atchley D.P., Yuan Y., Gutierrez-Barrera A., Hortobagyi G.N., Litton J.K., Arun B.K. (2011). Effectiveness of alternating mammography and magnetic resonance imaging for screening women with deleterious BRCA mutations at high risk of breast cancer. Cancer.

[17] Warner E., Messersmith H., Causer P., Eisen A., Shumak R., Plewes D. (2008). Systematic review: Using magnetic resonance imaging to screen women at high risk for breast cancer. Ann. Intern. Med..

[18] Riedl C.C., Luft N., Bernhart C., Weber M., Bernathova M., Tea M.K., Rudas M., Singer C.F., Helbich T.H. (2015). Triple-modality screening trial for familial breast cancer underlines the importance of magnetic resonance imaging and questions the role of mammography and ultrasound regardless of patient mutation status, age, and breast density. J. Clin. Oncol..

[19] Hartmann L.C., Sellers T.A., Schaid D.J., Frank T.S., Soderberg C.L., Sitta D.L., Frost M.H., Grant C.S., Donohue J.H., Woods J.E. (2001). Efficacy of bilateral prophylactic mastectomy in BRCA1 and BRCA2 gene mutation carriers. J. Natl. Cancer Inst..

[20] Meijers-Heijboer H., van Geel B., van Putten W.L., Henzen-Logmans S.C., Seynaeve C., Menke-Pluymers M.B., Bartels C.C., Verhoog L.C., van den Ouweland A.M., Niermeijer M.F. (2001). Breast cancer after prophylactic bilateral mastectomy in women with a BRCA1 or BRCA2 mutation. N. Engl. J. Med..

[21] Kauff N.D., Satagopan J.M., Robson M.E., Scheuer L., Hensley M., Hudis C.A., Ellis N.A., Boyd J., Borgen P.I., Barakat R.R. (2002). Risk-reducing salpingo-oophorectomy in women with a BRCA1 or BRCA2 mutation. N. Engl. J. Med..

[22] Kotsopoulos J., Huzarski T., Gronwald J., Singer C.F., Moller P., Lynch H.T., Armel S., Karlan B., Foulkes W.D., Neuhausen S.L. (2017). Bilateral Oophorectomy and Breast Cancer Risk in BRCA1 and BRCA2 Mutation Carriers. J. Natl. Cancer Inst..

[23] Domchek S.M., Friebel T.M., Singer C.F., Evans D.G., Lynch H.T., Isaacs C., Garber J.E., Neuhausen S.L., Matloff E., Eeles R. (2010). Association of risk-reducing surgery in BRCA1 or BRCA2 mutation carriers with cancer risk and mortality. JAMA.

[24] Domchek S.M., Friebel T.M., Neuhausen S.L., Wagner T., Evans G., Isaacs C., Garber J.E., Daly M.B., Eeles R., Matloff E. (2006). Mortality after bilateral salpingo-oophorectomy in BRCA1 and BRCA2 mutation carriers: A prospective cohort study. Lancet Oncol..

[25] Rebbeck T.R., Kauff N.D., Domchek S.M. (2009). Meta-analysis of risk reduction estimates associated with risk-reducing salpingo-oophorectomy in BRCA1 or BRCA2 mutation carriers. J. Natl. Cancer Inst..

[26] Lee E.G., Kang H.J., Lim M.C., Park B., Park S.J., Jung S.Y., Lee S., Kang H.S., Park S.Y., Park B. (2019). Different Patterns of Risk Reducing Decisions in Affected or Unaffected BRCA Pathogenic Variant Carriers. Cancer Res. Treat..

[27] Cheng H.H., Shevach J.W., Castro E., Couch F.J., Domchek S.M., Eeles R.A., Giri V.N., Hall M.J., King M.C., Lin D.W. (2024). BRCA1, BRCA2, and Associated Cancer Risks and Management for Male Patients: A Review. JAMA Oncol..

[28] Breast Cancer Linkage Consortium (1999). Cancer risks in BRCA2 mutation carriers. J. Natl. Cancer Inst..

[29] Leongamornlert D., Mahmud N., Tymrakiewicz M., Saunders E., Dadaev T., Castro E., Goh C., Govindasami K., Guy M., O’Brien L. (2012). Germline BRCA1 mutations increase prostate cancer risk. Br. J. Cancer.

[30] Castro E., Goh C., Olmos D., Saunders E., Leongamornlert D., Tymrakiewicz M., Mahmud N., Dadaev T., Govindasami K., Guy M. (2013). Germline BRCA mutations are associated with higher risk of nodal involvement, distant metastasis, and poor survival outcomes in prostate cancer. J. Clin. Oncol..

[31] DiSilvestro J.B., Haddad J., Robison K., Beffa L., Laprise J., Scalia-Wilbur J., Raker C., Clark M.A., Lokich E., Hofstatter E. (2024). Ovarian Cancer Risk-Reduction and Screening in BRCA1/2 Mutation Carriers. J. Women’s Health.

[32] Wainberg S., Husted J. (2004). Utilization of screening and preventive surgery among unaffected carriers of a BRCA1 or BRCA2 gene mutation. Cancer Epidemiol. Biomark. Prev..

